# Laparoscope-Assisted Guidance to the Pineal Region

**DOI:** 10.7759/cureus.9085

**Published:** 2020-07-09

**Authors:** Rose Fluss, Andrew J Kobets, David Altschul, Jonathan Nakhla, Patrick Lasala

**Affiliations:** 1 Neurological Surgery, Montefiore Medical Center/Albert Einstein College of Medicine, Bronx, USA; 2 Neurological Surgery, Montefiore Medical Center, Bronx, USA; 3 Neurological Surgery, Mobile Infirmary Medical Center, Mobile, USA; 4 Neurological Surgery, Albert Einstein College of Medicine, Bronx, USA

**Keywords:** abdominal laparoscope, endoscope, falcotentorial meningioma, pineal region, supracerebellar infratentorial approach, surgical ergonomics

## Abstract

Tumors arising in the pineal region present a number of challenges when planning for effective removal. This report describes the successful resection of a falcotentorial meningioma occurring in a 56-year-old female using a supracerebellar infratentorial approach.

In order to excise the pineal region mass, a unique combination of instrumentation was used, including a microscope, endoscope, and abdominal laparoscope.

This technique afforded us passage to the pineal region, which allowed for enhanced visualization and maneuverability and was more amenable to decreasing the physical stress of the operating surgeon.

This article is the first to detail the use of an abdominal laparoscope to remove a pineal tumor of this size for near-total resection. The various surgical approaches and tools traditionally used to remove pineal tumors are discussed, and the particular advantages and disadvantages of our hybrid approach are reviewed.

## Introduction

Falcotentorial meningiomas (FTM) comprise 8% of all pineal-region tumors and pose post-operative challenges due to their deep location and propensity to grow on either side of the tentorium [1-2]. Tumors can compress the pulvinar nuclei, tela choroidea, splenium of the corpus callosum, pineal gland, quadrigeminal plate, and tectum and can envelop the internal cerebral veins, the vein of Galen, and the straight sinus. Patients typically exhibit symptoms of increased intracranial hypertension, which can manifest as headaches, papilledema, nystagmus, tinnitus, postural instability, visual disturbances, memory impairment, and seizures. Injury to the dorsal midbrain can result in vertical gaze palsy or Parinaud’s syndrome [1,3]. FTMs commonly exhibit benign nature, which makes them more amenable to cure with gross total resection as compared to the other pineal-region tumors. Visualization and access still remain limitations to effective total resection [2].

Endoscopy provides a minimally invasive route to the pineal region and thus poses less threat of damaging neurovascular structures as compared to using an open microsurgical approach. Here, an endoscope functions like a telescope by providing magnification, in addition to a larger view, when using angled endoscopes and lenses. Being arm-level, endoscopy may also allow greater comfort to the operating surgeon. Given these advantages and disadvantages, our team developed a combined supracerebellar infratentorial approach using an operative microscope, endoscope, and, ultimately, an abdominal laparoscope for large falcotentorial meningioma resection. The combination of techniques allowed us to maximize surgical resection to the anterior margin of the lesion at a depth of 9 cm from the internal occipital protuberance while still providing judicious care to the vulnerable structures of this small region.

## Case presentation

Patient presentation

A right-handed, 56-year-old woman with a medical history significant for hypertension and obesity presented to the emergency room with ataxia and gait disturbances associated with frequent falls over a two-year period. She additionally complained of frequent diffuse headaches and a temporary, yet reversible, veil-like loss of vision in the right eye over the last few months. A subjective heaviness and dragging of the left leg were also noted. The patient was unable to ambulate more than 50 feet without stopping. On review of systems, including cognitive disturbance, additional visual disturbances, and urinary incontinence were absent.

On examination, she was an overweight middle-aged woman fully alert and oriented. Her papillary reflex was normal with intact extraocular movements, including the up- and down-gaze. Papilledema was noted. She ambulated with a walker but demonstrated full strength in her upper and lower extremities. Dysmetria and dysdiadochokinesia were noted bilaterally and pathological reflexes were absent.

**Figure 1:**
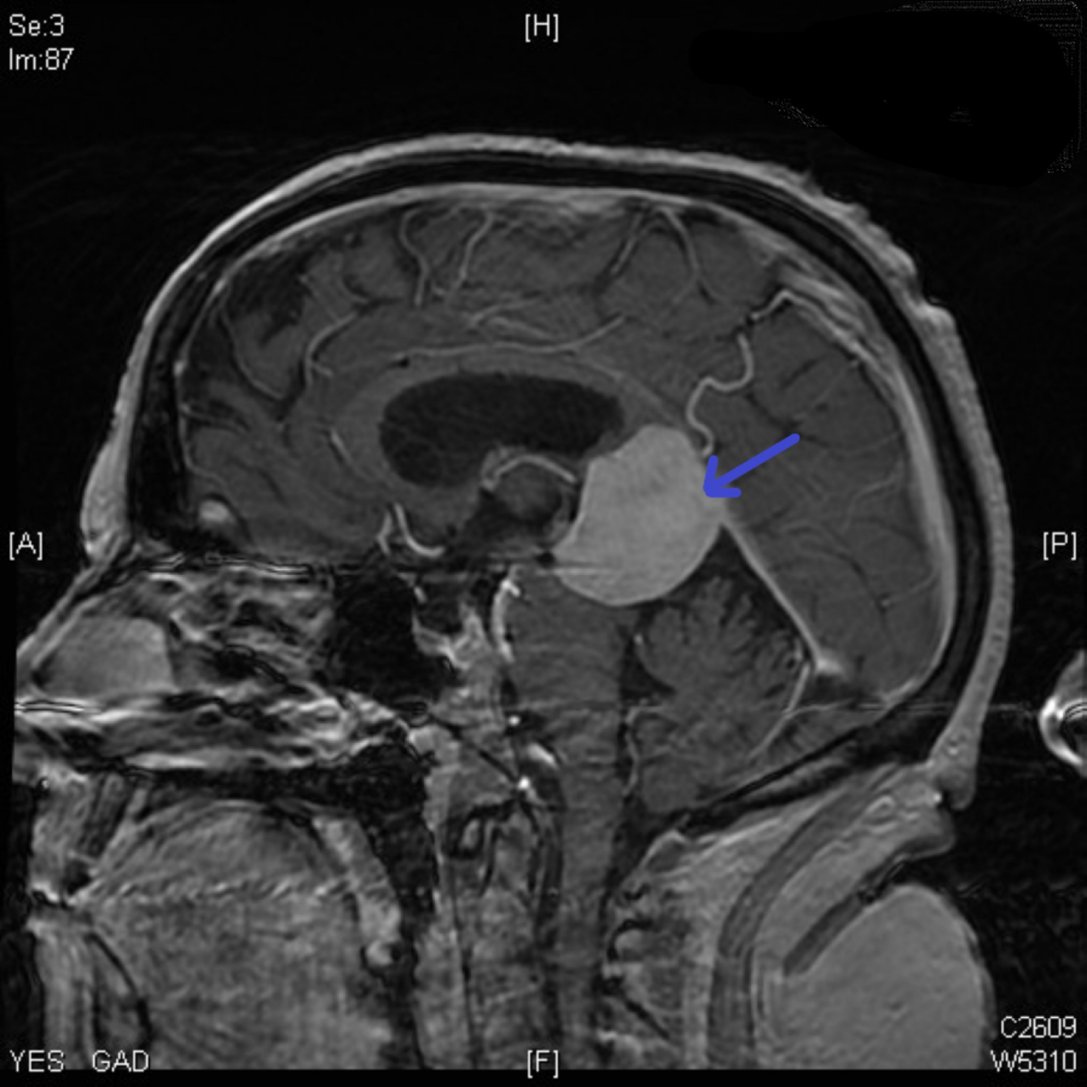
Preoperative MRI displaying the falcotentorial meningioma MRI: magnetic resonance imaging

Initial head computed tomography (CT) demonstrated a hyperdense midline mass at the falcotentorial border in the pineal region with a mass effect upon the cerebral aqueduct resulting in obstructive hydrocephalus. Magnetic resonance imaging (MRI) showed a 3.8 x 3.3 x 3.3cm extra-axial mass compressing the tectal plate, thalami, and splenium (Figure 1). The center of the lesion lied in a plane 43 degrees above the line adjoining the inion and the tip of the nose. Cerebrospinal fluid (CSF) tumor markers, including alpha-fetoprotein and beta-human chorionic gonadotropin, were negative, and CSF analysis did not demonstrate any malignant cells.

A ventriculoperitoneal shunt was placed to manage the hydrocephalus. The patient initially had a favorable clinical response but distal migration of the shunt occurred through a ventral hernia, and an abdominal abscess formed six weeks following her surgery. A decision was made to remove the shunt and a third ventriculostomy was performed without complication. In the following months, the patient’s headaches significantly improved and her gait difficulties had resolved. However, after six months, her headaches re-emerge insidiously, her gait was wide-based, and MRI demonstrated an increase in the size of her lesion. For these reasons, a decision was made at this time with the patient to undergo surgical resection of this lesion.

Operative technique

**Figure 2:**
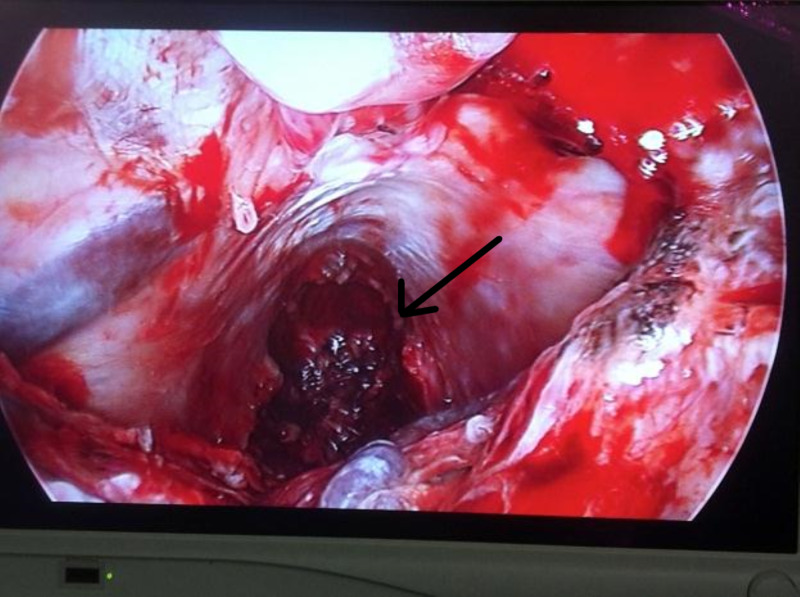
Intraoperative photograph depicting view using endoscopy

An endoscopically guided supracerebellar infratentorial surgical resection was planned and pre-operative, contrast-enhanced, thin-sliced MRI images were obtained and loaded into an intraoperative navigation system. In the operating suite, she was placed in a semi-sitting position with her neck gently flexed and an internal jugular venous catheter was placed to monitor for air emboli. The stereotactic navigation was used to guide a quadrangular bone flap bound superiorly by the transverse sinus after a midline occipital skin incision was made. The dura was opened with a Y-type incision and automatic vein clips were used for the occipital sinus. The operating microscope was then used to coagulate and divide the arachnoid adhesions and veins connecting the superior surface of the cerebellum and the tentorium allowing gravity to draw the cerebellum out of the operative field as these adhesions were lysed. The stereotactic probe was placed on the tumor surface and used to guide entry into the tumor capsule inferior and medial to venous structures on the lateral tumor surface. Microscissors were used to open the tumor and subcapsular resection was started using the microscope. Visualization was then transitioned to straight and 30-degree endoscopes along with elongated instruments, including various pituitary rings and curettes, in order to further facilitate resection (Figure 2).

**Figure 3:**
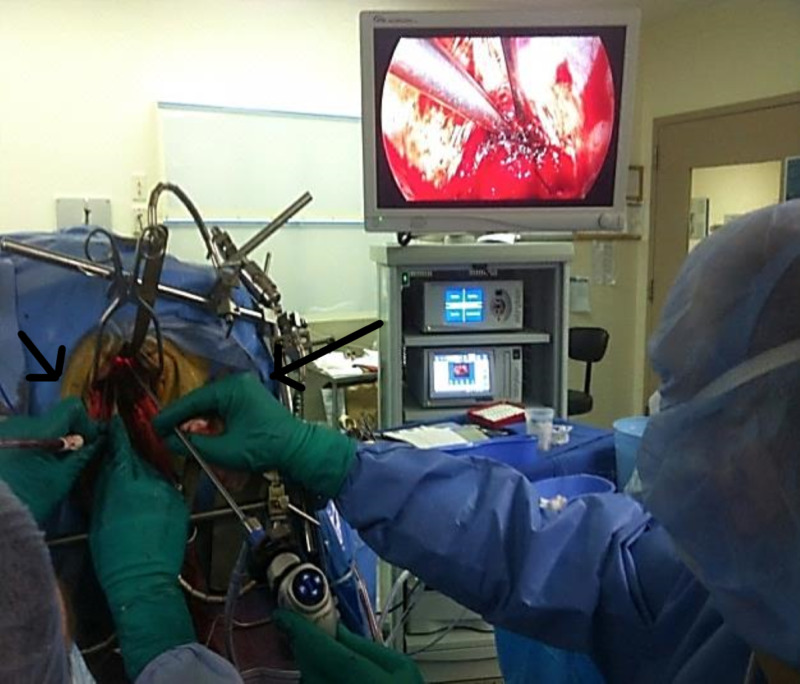
Dual operator approach to resection using endoscopy and laparoscopy.

When the limits of endoscopic guidance were reached, namely, when the assistant’s hands were directly abutting the patient’s skull, a laparoscope was obtained along with its instrument set in order to allow further reach into the depths on the lesion. While one operator guided the camera, the second placed traction on the lesion with one hand and dissected it with the other (Figure 3). At times, a four-handed technique was used, with a hand each utilized for the camera, suction, grasper, and bipolar cautery. Approach through burr holes may limit such four-instrument maneuverability. When the anterior capsule was reached, the straight laparoscope was transitioned to a 30-degree laparoscope and allowed for anterosuperior and anteroinferior access to the remaining portion of the tumor at its deepest margins. The surgeon’s hands were able to keep a distance from the cranial opening and were able to be stabilized with armrests. The longer length of the laparoscopes additionally allowed for greater angulation at the depth of exposure with less retraction of more superficial structures. After resection was completed, the surgeon was able to visualize the cavity with greater ease to ensure hemostasis was achieved.

**Figure 4:**
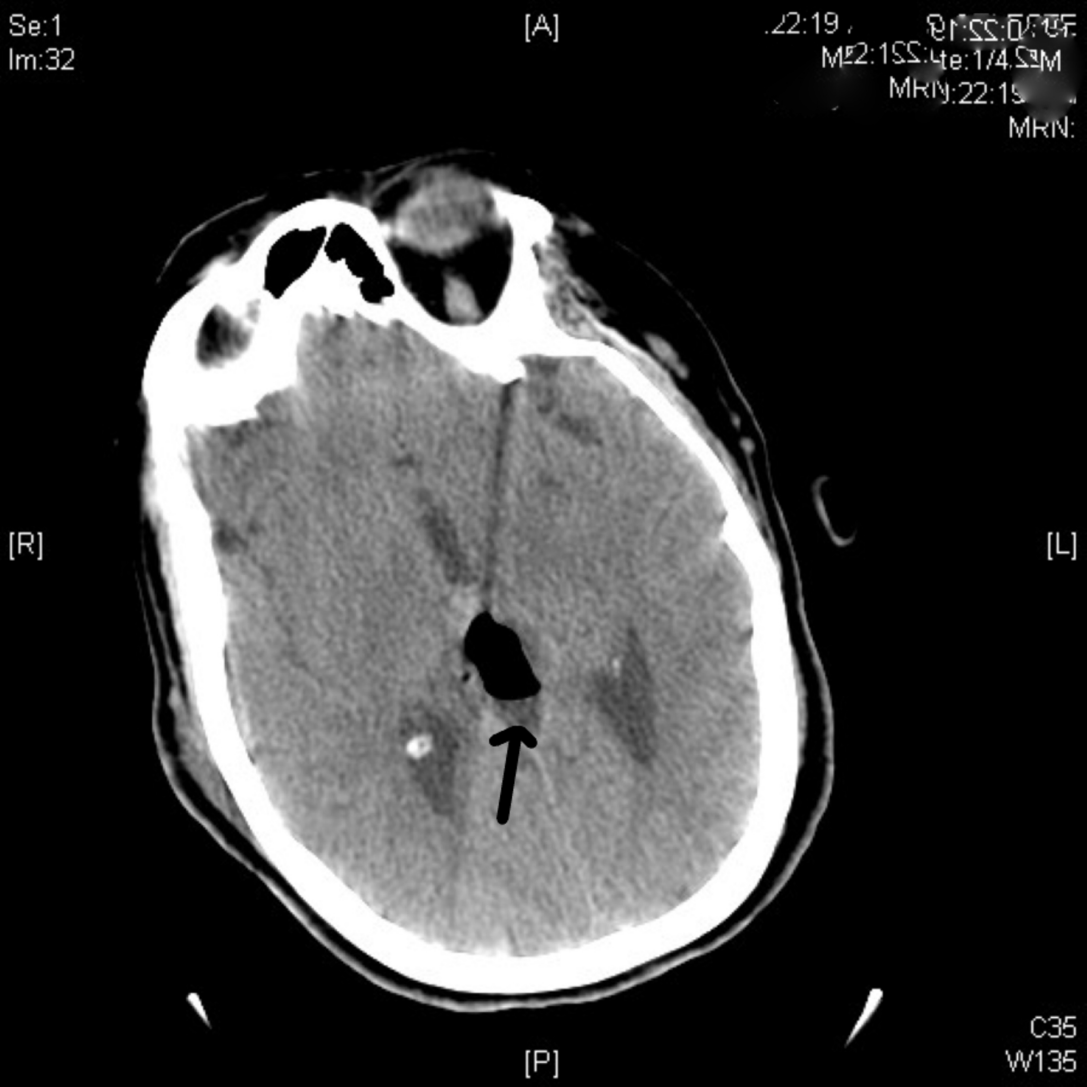
Axial CT scan showing postoperative pseudomeningocele CT: computed tomography

**Figure 5:**
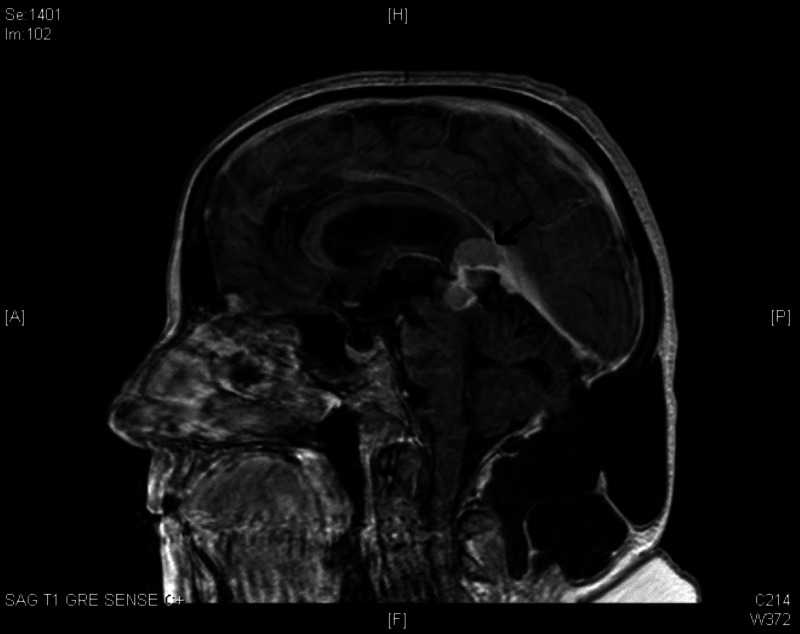
Postoperative MRI showing a small remnant tumor with pseudomeningocele MRI: magnetic resonance imaging

The patient tolerated the procedure and underwent an uneventful postoperative course after which she was discharged to acute rehab on postoperative Day 5. Her ambulation improved to 300 feet with the assistance of her cane and her strength improved throughout. She complained of no visual disturbances and was discharged home after 10 days of rehabilitation. Postoperative CT and MRI (Figures 4, 5) showed a pseudomeningocele but the patient never developed a CSF leak, and it was managed expectantly. Pathologic examination of the tumor demonstrated moderately hypercellular, palisading spindle cells, with ill-defined whorl formations and a MIB1/Ki-67 proliferation index variable ranging up to 15%. Progesterone receptors were seen in approximately 50% of the tumor cell nuclei. A diagnosis of a World Health Organization (WHO) grade II meningioma was made based on these findings. The patient underwent radiation therapy for the small residual tumor and remained clinically stable without tumor growth at the six- and 12-month follow-ups. Her pseudomeningocele resolved without intervention.

## Discussion

Surgical options to the pineal region include the transventricular, suboccipital transtentorial, supracerebellar infratentorial, transcallosal, and transparietal approaches. Each has advantages and disadvantages and taken with tumor-specific factors, define an optimal approach.

The suboccipital transtentorial approach allows for the visualization of larger, more superior lesions in the tentorial hiatus, with excellent views of the tectum and tentorial notch. However, the need for occipital retraction results in postoperative visual complications in a majority of patients, namely, homonymous hemianopsia immediately postoperatively [4]. Longer follow-up may demonstrate persistent visual field deficits in addition to pupillary light/near dissociation, up-gaze paresis, and convergence or retraction nystagmus. The risk of damaging the deep venous structures upon incising the tentorium is also present and the obliqueness of the approach complicates anatomic dissection within the surgical cavity, which in itself may necessity further occipital retraction.

The supracerebellar approach is optimal for small lesions below the cerebral veins and requires minimal cerebral retraction. It was first described by Krause in 1913, applied by Stein in 1979, who established it as a dependable route to the pineal region because it is a more direct midline route to the pineal region, which places less risk to neurovascular structures and allows a large tumor to sink into the operative field during resection [5]. However, the patient must be prone or in the sitting position, each of which presents with its own unique disadvantages. For example, the sitting position is known to increase the risk of air embolism and can be an uncomfortable and tiring position for operating surgeons. The prone approach has a risk for blindness from ischemic optic neuropathy, has blood pool into the operative field, and can be difficult in situations with a steep tentorial angle. A steep tentorium additionally precludes the optimal visualization of lesions, leaving the tentorial apex and the superior tectal edge relative blind spots in this approach. Complete anterior and superior resection is further limited by the length and angulation of available neurosurgical instruments.

With the development of neuroendoscopic techniques, endoscopy was applied in 1996 to the resection of two symptomatic quadrigeminal plate cysts [6]. Gore et al. provided a more detailed analysis of endoscopic supracerebellar cyst fenestration in 2008 [7]. In 2011, the first report of a pineal tumor resection purely using a mounted suction endoscope was made [8]. Tseng et al. and Mamelak et al. in 2013 were each the first to describe tumor resection in the pineal region using an endoscopic supracerebellar infratentorial approach [9-10]. Limitations to alternate approaches, such as the parapineal zone, have been cautioned by Zanini et al. whose patient experienced lasting upward gaze palsy postoperatively [11]. In 2015, Zaidi et.al quantified optimal variants of the supracerebellar approach based on the region one wishes to target. For the pineal region, they found that when using an endoscope, the lateral approach provided the greatest surgical freedom of 34.5 cm^2^ but the extreme lateral approach provided the largest horizontal angle of attack at 14.5° [12]. In recent years, the use of an exclusively endoscopic technique in the pineal region was described by Gu et al. and, later, Snyder et al. who report using a purely endoscopic and endoscope-assisted paramedian supracerebellar infratentorial approach to successfully operate on two patients presenting with pineal-region tumors [13-14].

Our case represents the largest tumor resection of the quadrigeminal cistern to date using a hybrid open/endoscopic/laparoscopic technique, necessitated by intraoperative challenges faced as we reached the limits of our standard neurosurgical instruments. The use of a laparoscopic instrument set afforded us better visualization and increased surgeon comfort, therefore, allowing for safer resection when compared to either the endoscopic or the microsurgical approach alone. The surgeon chose to utilize the laparoscope intraoperatively because the positioning of the endoscope was reaching its limit of depth, as it was brought to the dural edge upon deep resection of the lesion. The shorter length of the endoscope made the assistant hold their hands high at the patient's head in an awkward position, with limited access to the depths of the exposure. Therefore, the laparoscope was brought into the case to provide a more ergonomic manner for the assistant to hold the scope, whose camera could be brought right to the surface of the lesion; this was not possible with the endoscope. During the hours that ensued to resect the remainder of the lesion, fatigue became less of a factor utilizing the laparoscope for the assistant, and the surgeon was granted a highly magnified view of the lesion even at its farthest depths.

As we become more familiar with these hybrid approaches, we may expand their use to treat larger and more complex lesions, which would otherwise not be safely amenable to purely microsurgical or endoscopic resection. In addition, given the wide availability of these laparoscopic instruments, their use could readily supplement the standard surgical armamentarium for any pineal region resection or cyst fenestration.

While neuroendoscopes come in a variety of sizes for the management of a myriad of cranial pathology, rarely is the length of the lesions a substantial consideration in the choice for endoscopes in this field. Namely, and especially in children, the diameter of the endoscope that enters the ventricular system through a burr hole, or the nasal cavity to reach the sella turcica, is more important, and the smaller the diameter of the scope, the more useful it is for certain approaches. Endoscopes can range from 1.7 mm to 8 mm in diameter and the point of entry will determine the optimal diameter size. However, length is rarely a limiting factor in neuroendoscopy, and, as a consequence, endoscopes are typically 6, 10, or 11 cm long. For a lesion 9 cm from the dura, it would be unlikely that these instruments would be sufficiently long for a surgeon’s hands to be comfortable and not immediately at the patient’s skull in an uncomfortable, extended position. While longer endoscopes exist, namely, between 18 and 24 cm long, these are rarely readily available in the hospital as part of a basic neuroendoscopy set. However, laparoscopes are widely available throughout each hospital and can be utilized in any given moment of a surgical procedure to provide benefit to the surgeon as described. Typical laparoscopes can range in size from 26 cm to up to 50 cm. A broader range of these devices are expected to be available to the general surgeon and, thus, the neurosurgeon-in-need. Therefore, there is a substantial benefit of these instruments to be directly facing deep-seated lesions, allowing a surgeon's hands to be comfortably away from the skull, to facilitate their maneuverability and control over hours. Therefore, there is a profound impact these readily available instruments can have for the surgeon, and as this report promotes, they must be aware of their availability to provide the best outcomes for their patients.

The limitations of purely endoscopic supracerebellar techniques for the quadrigeminal cistern, as previously published by Tseng et al. and Gore et al., include the limited maneuverability in a region housing larger lesions, the limited visibility beyond the roof of the third ventricle and upper tectal region, the limited utility and length of endoscopic forceps, graspers, curettes, and probes (especially with hard lesions), and the significant challenges of hemorrhagic control under two-dimensional scope visualization, which could necessitate open conversion when vigorous bleeding is encountered [7,10]. The use of a purely endoscopic technique is limited to small or cystic lesions and would have been contraindicated for the lesion present in our current report.

As compared with standard endoscopic transsphenoidal or transventricular approaches that have a narrow corridor of entry, access to the pineal region is solely dependent on the degree of bone removed and the amount of relaxation afforded by the intervening tissue. In our case, we had improved cephalad visualization compared to prior reports because we used 30° and 45° endoscopes. Seeing to the corners of the quadrigeminal cistern can be greatly improved with an endoscope allowing one to see areas you could never see using a microscope, which only allows line of sight. The increased visualization makes the operations safer and more comfortable for the surgeon. In addition, we had improved maneuverability with the scope due to the large craniectomy performed.

Our hybrid approach addresses the limitations of the purely endoscopic approach in the following manners: the initial microsurgical dissections provide a larger corridor through which instruments can be maneuvered to a greater degree, the limited reach of the endoscope is supplanted by the superior field of view of the laparoscope, the open approach allows for several instruments to be placed in the resection cavity, which is not simply bound by a number of burr holes placed, and, finally, an open exposure with long instruments allows for the opportunity of greater hemostatic control, if necessary, during the course of the surgery. These longer instruments can also allow for greater visualization, control, and comfort for the surgeon over the hours of the surgical procedure.

Despite these advantages, our technique has inherent limitations as well. For example, the far inferior and anterior border of the tectum remains a blind spot, as does the superior-most portion of the underside of the tentorium. Since the patient is placed in the seated position, there is also a threat of air embolism. Additionally, there may be a greater risk of a CSF leak with a full craniotomy as compared to the burr holes required for a purely endoscopic approach. Finally, a laparoscope requires a surgeon to have experience using it.

While total resection is theoretically possible with 30° laparoscopes directed upward to the tentorium and downward to the tectum, a risk to these structures remains. The danger lies as a consequence of compression resulting from extreme angulation of our cameras and instruments, as well as with microdissection of the lesion off of vulnerable structures such as the brainstem. Therefore, practice with these new laparoscopic visualization options is necessary for the safe resection of large lesions in the falcotentorial region.

## Conclusions

While falcotentorial meningiomas are rare, their benign nature and relative size result in unique surgical challenges. The supracerebellar approach has been used for decades to resect these lesions, however, limits are encountered when attempts are made to reach deeper within these lesions. A recent trend toward endoscopically guided supracerebellar resection of pineal cysts and small tumors has been described, however, to date, no attempts have been made to utilize this approach for using an abdominal laparoscope. Our development of a hybrid open/endoscopic/laparoscopic technique has allowed for the depth necessary to resect such large lesions, with the ease and control of the surgeon being at a comfortable length away from the skull. While total resection is feasible given the utility of this novel approach, subtotal resection is still favored in order to protect vital structures that may be adherent to the tumor itself. Further work should evaluate the outcomes of these methods as they compare to the surgical outcomes of alternative approaches for lesions of this size. Additionally, work should focus on the development of intraoperative camera and instrument positioning systems in order to minimize surgeon strain during these long and intricate procedures. Finally, the surgeon must be aware that their instrument sets are not limited to only neurosurgical instruments and that considering the possibility of a laparoscopy-assisted approach can provide better outcomes for their patients.
